# Enrichment of Lactoferrin and Immunoglobulin G from Acid Whey by Cross-Flow Filtration

**DOI:** 10.3390/foods12112163

**Published:** 2023-05-26

**Authors:** Fabian Ostertag, Jörg Hinrichs

**Affiliations:** Department of Soft Matter Science and Dairy Technology, Institute of Food Science and Biotechnology, University of Hohenheim, Garben Str. 21, 70599 Stuttgart, Germany; fabian.ostertag@uni-hohenheim.de

**Keywords:** ultrafiltration, dairy processing, whey protein concentrate, acid whey, biofunctional protein

## Abstract

The production of cream cheese, curd, high-protein yogurt, or caseinate results in large amounts of acid whey as a by-product. So far acid whey is often disposed as animal feed or organic fertilizer. However, these approaches ignore the valorization potential that arises from the unique composition of the whey protein fraction. Whey contains the biofunctional proteins lactoferrin and immunoglobulin G, which possess immune-supporting, antibacterial, antiviral, and numerous further health-promoting functions. However, the concentration of these proteins in bovine milk or whey is below a physiologically relevant level. Based on literature research we specified a daily intake of 200 mg lactoferrin as the minimal functional dose. By means of cross-flow ultrafiltration, an attempt was made to increase the concentration of biofunctional proteins. Therefore, a membrane for the selective retention of lactoferrin and immunoglobulin G was identified, and the process parameters were optimized. Finally, a concentration experiment was conducted, whereby the concentration of biofunctional proteins was increased up to factor 30. The biofunctionality was assessed in a microbiological assay. Surprisingly, the antimicrobial growth inhibition of the produced concentrate was even higher than in pure lactoferrin. The presented approach offers a strategy to convert an abundant but underutilized by-product into valuable products for human nutrition.

## 1. Introduction

The production of cheese, high-protein yogurt, and caseinate results in large amounts of whey as a highly abundant by-product. Based on an approximated number of 200 million tons per year, this means that nearly one-quarter of worldwide milk productions is converted to whey [1]. In general, this whey fraction contains approximately 55% of the nutrients from milk, including 20% of the total protein content [2]. Depending on the principle of coagulation it can be distinguished into sweet- (enzymatically induced coagulation) and acid whey (pH-induced coagulation). While dried sweet whey powder has become a highly demanded trade product (e.g., ingredient for nutrition bars, beverages, infant formulas, bakery items, soups, or dairy products), the valorization of acid whey remains challenging. Due to the dominant acidic taste, its use as a bulk ingredient in food applications is restricted. Furthermore, the low pH (between 4.0 and 5.0) and high mineral content (6–8 g·L^−1^) impede the processing with conventional unit operations such as evaporation, filtration, or spray drying [3].

Therefore, acid whey is often used as animal feed, organic fertilizer, or as a substrate for biogas production. These approaches might allow cost-neutral disposals, but they ignore the valorization potential that arises from the unique composition of the whey protein fraction. Besides the major whey proteins, β-lactoglobulin (β-Lg) and α-lactalbumin (α-La), biofunctional whey proteins such as lactoferrin (Lf) and immunoglobulins (Ig) can be found in whey [4]. The whey protein fraction is dominated by β-Lg (c_β-Lg_ ≈ 3–4 g·L^−1^) and α-La (c_α-La_ ≈ 1.5 g·L^−1^), which contribute with more than 70% to the total protein content. In contrast, the concentrations of Lf (c_LF_ ≈ 0.03–0.2 g·L^−1^) and Ig (c_Ig_ ≈ 0.3–0.6 g∙L^−1^) are notably lower [4,5]. Due to the heat sensitivity of whey proteins, the actual protein content can vary greatly. The denaturation temperature of whey proteins starts at 60 °C [6]. Depending on the selected temperature-time combination, considerable losses of native whey protein may occur. While pasteurization results in only marginal denaturation losses (at 72 °C for 30 s we observed a loss of approximately 10%), intensive heating (e.g., 90 °C for 5 min), as it is partly applied in the production of fermented milk products, leads to a complete loss of native whey protein. Therefore, gentle process conditions are essential for the optimal valorization of whey proteins.

**Figure 1:**
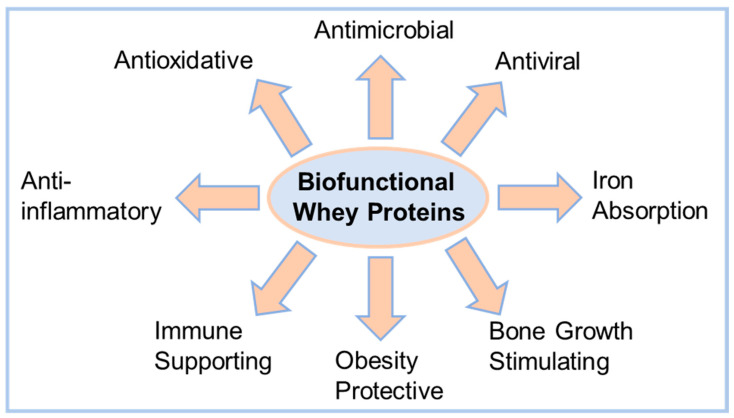
Overview of the manifold biofunctional activities of minor whey proteins. The figure sums up biofunctional properties of bovine lactoferrin and immunoglobulin proteins as described in the literature.

Lactoferrin is an 80 kDa glycoprotein, which is involved in iron metabolism and numerous other biological functions as presented in Figure 1. For newborn infants, the supportive effects of this protein on the immune system are vital for the first few days following birth. This specific role is emphasized by the increased Lf content in colostrum, the first milk following delivery of the newborn. In human colostrum, Lf is a highly abundant protein, with concentrations up to 4.5–15.3 g·L^−1^, while in mature human milk the concentration decreases to 1.4–2.8 g·L^−1^ [7]. A similar progression can be found in bovine colostrum (c_Lf_ = 0.8–2.0 g·L^−1^) and mature bovine milk (c_Lf_ = 0.03–0.2 g·L^−1^) [5]. Lönnerdal et al. [8] have demonstrated that human and bovine Lf show a large sequence homology and Lf of both species can be bound by human Lf receptors, which are located in the small intestine. Consequently, bovine Lf is an important ingredient in infant formulas since these formulas try to mimic the natural composition of human milk. However, studies showed that not only infants can benefit from Lf supplementation. The iron absorption enhancing properties [9,10,11] or the antiviral and antibacterial activities bear the potential of health-promoting properties for people of all ages. Well-described are the versatile antimicrobial properties of Lf, which can be divided into antibacterial [12] and antifungal [13] activities. In recent years, the antiviral effect against coronaviruses has been of particular interest and has been studied in detail from cell culture studies [14] to clinical trials [15]. In addition, there are indications for numerous further host-defensive effects, for example against common viral infections (e.g., influenza, herpes, noro- or rotaviruses) [16]. Figure 1 summarises a number of further biofunctional characteristics of Lf, including bone growth stimulating [17,18] or obesity protective properties [19,20], as well as immune supporting [21,22], anti-inflammatory [23] and antioxidative effects [24].

Because of these unique properties, Lf has been isolated from milk and sweet whey using cation-exchange chromatography on an industrial scale since the mid-1980s [25]. The global market for this high-value protein was estimated at 236 million USD in 2021, while sales of over 400 million USD are forecast for 2028, underlining the increasing growth rate for this market segment [26]. In the European Union, the use of bovine Lf as a food ingredient or nutraceutical is regulated by the novel food regulation [27], which allows the incorporation of Lf in dairy- and non-dairy-based food products and beverages. While infant formula remains the most common application, new areas of use such as the use of Lf powder as a dietary supplement, food ingredient (e.g., in yogurt, chewing gums, or beverages), pharmaceutical, cosmetics or animal nutrition are steadily gaining ground [26,28].

Immunoglobulins (also referred to as antibodies) are further high-value proteins, which are present in acid whey. In general, immunoglobulins can be divided into five classes (IgG, IgA, IgM, IgD and IgE). In bovine milk and whey IgG is the dominating class with a share of approximately 90% of the total immunoglobulin content. In analogy to Lf, elevated Ig concentrations were measured in colostrum (human: 7.7–59.3 g·L^−1^; bovine: ~58.6 g·L^−1^), compared to mature milk (human: 0.8–1.4 g·L^−1^; bovine: ~0.8 g·L^−1^) [7,29]. Immunoglobulins can recognize specific molecular patterns of foreign cells or viruses, resulting in antigen–antibody reactions. It was demonstrated that bovine IgG can bind to many human pathogens and allergens. In clinical studies, a preventive effect on gastrointestinal- and respiratory tract infections and inflammations was shown. The specific antibody–antigen interaction offers the opportunity to derive IgGs from hyperimmunized cows, allowing a targeted treatment of infectious diseases as for instance against rotavirus infections in infants and children [30,31]. However, health-promoting properties have also been described in the literature for IgG from non-immunized cows. In a comprehensive review, Ulfman et al. [21] describe the supportive effects of bovine IgG for gastrointestinal and respiratory tract infections, as well as for inflammation reactions.

As shown in Figure 1, the biofunctional proteins Lf and IgG show preventive, therapeutic, or therapy-supporting effects against numerous widespread diseases and deficiencies (e.g., iron deficiency, obesity, infections, bone resorption). However, the concentration of biofunctional proteins in bovine milk or whey is low, which makes it almost impossible to take advantage of the discussed health benefits in a normal diet. Although clear dose–response relationships are not established yet, there is literature-based evidence for health-promoting effects at a minimal daily intake of 200 mg Lf, since this dose showed significant effects in clinical trials. Nappi et al. [9] observed in a randomized clinical study, conducted in pregnant women (*n* = 100), significant improvements in hemoglobin and serum ferritin levels. The daily dose in this study was 200 mg Lf over a period of 30 days. Further clinical trials confirmed these effects [10,11], and this dose has also been shown to have a demonstrable effect in other conditions such as obesity [19] or COVID-19 infections [32]. At the current time, this daily intake can be only covered by the consumption of supplements, containing purified Lf powder. Therefore, Lf is isolated and purified from milk or sweet whey by cation-exchange chromatography [25]. This process results in a Lf powder of high purity (>95%), but requires high expenditures in regard to costs, resources and energy. 

The objective of the following study is the enrichment of the biofunctional proteins Lf and IgG in acid whey. It is hypothesized that the initially low concentration of these proteins can be increased by means of cross-flow ultrafiltration to a physiologically functional level, comparable to human milk. Membrane filtration is a cost- and resource-efficient unit operation, which is already widely applied for dairy processing. Furthermore, the gentle and product-preserving process conditions will help to keep the proteins in their native and functional state. This approach would help to convert acid whey, a so far underutilized by-product, into a valuable and affordable nutritional supplement.

## 2. Materials and Methods

### 2.1. Acid Whey Production and Clarification

To ensure a standardized composition, the acid whey was produced in the pilot dairy for research and training (University of Hohenheim, Stuttgart, Germany) prior to the filtration experiments. The raw milk was obtained from the research station Meiereihof (University of Hohenheim, Stuttgart, Germany). After arrival at the dairy plant, the milk was separated into cream and skim milk and pasteurized. In contrast to more intensive thermal treatments, the moderate pasteurization conditions (ϑ = 72 °C for 30 s) resulted only in a marginal decrease (approximately 10%) of the protein concentration in whey. For whey production, 1000 L of pasteurized skim milk were fermented for 16 h at 22 °C using 100 g of a mesophilic starter culture (Probat 505, DuPont de Nemours Inc., Wilmington, DE, USA). When a pH of 4.5 was reached the curd was filled into linen filter bags and the drained whey was collected in a basin.

To remove microorganisms (e.g., starter culture and contaminants) and cheese fines, the collected whey was clarified by microfiltration. Therefore, a 1.4 µm ceramic membrane with an active area of 1.69 m^2^ (Membralox 3258 4C, Pall Corp., Port Washington, NY, USA) was used. The clarification was conducted at 8 °C with a transmembrane pressure (Δp_TM_) of 0.1 MPa and an averaged cross-flow velocity of 7.0 m·s^−1^. Under cooled conditions (6 °C) the clarified acid whey showed a good shelf life, enabling storage for 7 days in a stack tank. This allowed the accumulation of 800 L of acid whey for the concentration experiment. 

#### 2.1.1. Filtration—Calculations and Characteristic Numbers

The underlying objective for the filtration runs was the efficient and selective enrichment of the proteins Lf and IgG in acid whey. Therefore, a high concentration factor (CF_Lf/IgG_) for these specific proteins was endeavored. To differentiate between the concentration factor of an individual component (referred to as Cfi), the abbreviation CF_Volume_ is used for the volume concentration factor. For the description and comparison of the filtration progress, the following numbers were applied: 

The transmembrane pressure (Δp_TM_, Equation (1)) describes the averaged pressure difference over the full membrane length between the retentate and permeate side. The axial pressure difference (Δp_axial_, Equation (2)) describes the pressure drop due to frictional forces between the membrane in- and outlet on the retentate side:

(1) $$△p_{\mathrm{TM}}=\frac{p_{\mathrm{inlet}}+{\;p}_{\mathrm{outlet}}}{2}-p_{\mathrm{permeate}}$$

(2) $$△p_{\mathrm{axial}}=p_{\mathrm{inlet}}-p_{\mathrm{outlet}}$$

The Δp_TM_ is the driving force for the permeate flux (J_Permeate_, Equation (3)), which is a comparable measure for filtration efficiency. It describes the permeate volume flow ($\dot{V}$_Permeate_) within a normalized membrane area (A):

(3) $$J_{\mathrm{Permeate}}=\frac{{\dot{V}}_{\mathrm{Permeate}}}{A}$$

Concerning the retention characteristics, the transmission of a certain component i (Tr_i_; Equation (4)) is a conclusive number, describing the transfer of a component through the membrane (the term “permeation” can be used synonymously). The calculation is based on the concentration quotient of the corresponding component (c_i_) in the permeate and retentate. Vice versa, the retention of component (Ret_i_; Equation (5)) can be calculated:

(4) $${\mathrm{Tr}}_{i}=\frac{c_{i,\mathrm{Permeate}}}{c_{i,\mathrm{Retentate}}}$$

(5) $${\mathrm{Ret}}_{i}=1-{\mathrm{Tr}}_{i}$$

When a component is permeating through the membrane (Tr_i_ > 0), the mass flux for this specific component (J_i_, Equation (6)) can be calculated by combining c_i,Permeate_ and J_Permeate_:

(6) $$J_{i}={\;J}_{\mathrm{Permeate}}\cdot c_{i,\mathrm{Permeate}}$$

To meet the initially defined objectives, a high J_Permeate_ and a full retention of Lf and IgG (Tr_Lf/IgG_ = 0) was aspired. At the same time, a high transmission of the highly abundant whey proteins α-La and ß-Lg was desired.

#### 2.1.2. Filtration—Plant Design and Membrane Selection

The filtration runs were conducted on a batch pilot plant (MMS Membrane Systems AG, Zurich, Switzerland) using a 3.8″ spiral wound membrane module. The filtration tank was equipped with a 500 L feed tank, two centrifugal pumps (feed and circulatory pump) and a tubular heat exchanger (a detailed PI-diagram is attached as Supplementary Material). The pressure was regulated by the frequency of the feed pump and manually adjustable control valves. Sensors recorded the feed temperature, volume flows, and pressure settings during the experiments. Depending on the intention of the experiment, the filtration can be distinguished into equilibrium and concentration mode. While in the concentration mode, the permeate was continuously removed from the feed, resulting in a concentration of the retained components, the permeate was recycled back to the feed in equilibrium mode. The advantage of the equilibrium mode lies in the consistent feed composition, allowing a direct comparison of filtration properties unaffected by feed alterations, as they unavoidably occur in concentration mode. 

To find the ideal membrane for the targeted enrichment of Lf and IgG, 3 spiral-wound modules with membranes in the ultra- and microfiltration range were compared. The following membranes were used:

- Koch K131 (MWCO: 10 kDa, area: 6.7 m^2^, spacer: 31 mil, Koch Separation Solutions, Wilmington, MA, USA)
- Trisep UB50 (nominal pore size: 30 nm, area: 5.4 m^2^, spacer: 46 mil, Microdyn-Nadir GmbH, Wiesbaden, Germany)
- Nadir MV020 (nominal pore size: 200 nm, area: 5.4 m^2^, spacer: 46 mil, Microdyn-Nadir GmbH, Wiesbaden, Germany)

After each filtration run, membranes were rinsed with distilled water and cleaned using alkaline (0.2% P3-Ultrasil 112, Ecolab Inc., Saint Paul, MN, USA) and acidic (0.2% Ultrasil 75, Ecolab Inc., Saint Paul, MN, USA) cleaning solutions. Each treatment was performed for at least 30 min at 50 °C. The cleaning success was confirmed by the recovery of the initial water flux values.

#### 2.1.3. Filtration—Parameter Setting and Optimization of Transmembrane Pressure

The objective of the initial filtration experiment was the selection of an appropriate membrane. Therefore, 200 L of acid whey were filtered for 100 min at 50 °C in equilibrium mode. The transmembrane pressure (Δp_TM_) was set to 100 kPa at a volume flow of 4000 L·h^−1^, corresponding to a cross-flow velocity of 0.36 m·s^−1^ (Trisep UB50, Nadir MV020) or 0.41 m·s^−1^ (Koch K131), respectively. Samples from the permeate and retentate stream were taken after 5 and 100 min during equilibrium mode. These sampling times were chosen because after 5 min the deposit layer is at the beginning of the build-up phase (due to technical limitations an earlier sampling was not possible), while after 100 min a state of equilibrium is reached. This initial filtration experiment was conducted in triplicate for each membrane.

For all subsequent filtration runs the Trisep UB50 membrane was used in single determination. Besides the membrane, numerous further parameters affect the filtration characteristics during cross-flow filtration. Directly controlled can be the temperature (ϑ), cross-flow velocity (w), and Δp_TM_. To avoid microbial growth and denaturation of heat-sensitive whey proteins the temperature was set to ϑ = 8 °C. The cross-flow velocity was set to w = 0.58 m·s^−1^, resulting in an axial pressure drop of Δ_paxial_ = 100 kPa. A higher cross-flow velocity results in increased shear rates and less deposit layer formation on the membrane surface; however, this parameter is limited by the stability of the membrane module. For most spiral wound modules, the maximum Δp_axial_ is specified with 100 kPa; consequently, the scope for further enhancements of this parameter is limited. 

Furthermore, attention must be paid to Δp_TM_ since it is the driving force for all ultra- and microfiltration applications. A higher Δp_TM_ leads to increased flux values, but also to an intensified and more compressed deposit layer. Consequently, the increase of Δp_TM_ leads only up to a certain limit, which is referred to as critical Δp_TM_, to a higher permeate flux [33]. In addition, the transmission of the smaller proteins might be affected by the Δp_TM_ [34]. To find the optimal Δp_TM_ seven individual filtration runs with different pressure settings (Δp_TM_ = 20, 60, 100, 140, 180, 220 and 260 kPa) were conducted. For each run 120 L of clarified acid whey were used and concentrated up to a CF_Volume_ of 5. Samples of the permeate and retentate were collected directly after filtration start (at CF_Volume_ = 1) and at CF_Volume_ = 2 and 5.

#### 2.1.4. Filtration—Enrichment of Biofunctional Proteins

Based on the optimized Δp_TM_ (Section 2.1.3) a biofunctional whey protein concentrate (WPC_biofunct._) with increased Lf and IgG content was produced. For this final filtration run a total volume of 800 L clarified acid whey was used. Due to limited storage capacities during the accumulation phase, the whey was pre-concentrated (CF_Volume_ = 2) prior to the production of WPC_biofunct._ During the concentration run all filtration parameters were kept constant (Δp_TM_ = 100 kPA; ϑ = 8–9 °C; w = 0.58 m·s^−1^). Retentate and permeate samples were collected at CF_Volume_ = 2, 4, 8, 16, 24, and 30.

### 2.2. Analytical Methods

The total nitrogen content in all permeate and retentate samples was quantified according to the Dumas method using a nitrogen analyzer (Dumatherm, Gerhardt GmbH & Co. KG, Königswinter, Germany) [35]. For the calculation of the overall crude protein content, a nitrogen-to-protein conversion factor of 6.38 was used. The dry matter content was determined with the drying oven method, according to [36].

The quantification of the whey proteins α-La, β-Lg and Lf was performed by reversed-phase HPLC. Therefore, an Agilent 1200 series unit consisting of a vacuum degasser, quaternary pump (flow rate 0.8 mL·min^−1^), autosampler (injection volume 20 µL), temperature-controlled column compartment (40 °C), and a DAD-detector (210 nm) was used (Agilent Technologies Inc., Santa Clara, CA, USA). As stationary phase served the BioResolve RP mAb Polyphenyl 450A column (dimension: 4.6 × 150 mm, particle 2.7 µm; Waters Corp, Milford, MA, USA). The mobile phase was composed of double distilled water (eluent A) and acetonitrile (eluent B). Trifluoroacetic acid (0.1%) served in both eluents as ion-pairing reagent. The elution was performed in a stepped gradient mode, a comprehensive method description is given by Ostertag et al. [37]. Calibration standards for each protein were purchased by Merck kGaA (Darmstadt, Germany).

For the quantification of bovine IgG, a commercial ELISA assay (Bovine IgG 3150-1HD-6, Mabtech Inc., Cincinnati, OH, USA) was used. For an improved linear calibration range, the calibrators were evenly distributed between 10 and 70 ng·mL^−1^. Before the analysis, all samples were diluted with sample buffer within the calibrated working range.

The viscosity at 20 °C was measured by a rotational rheometer (Physica MCR301, Anton Paar Group AG, Graz, Austria) using a cylindrical double gap geometry (DG27, Anton Paar Group AG, Austria; Dimensions Bob: length = 40 mm, d_inner_ = 25.003 mm, d_outer_ = 27.002 mm; Dimension Cup: d_inner_ = 23.047 mm, d_outer_ = 29.278 mm). Therefore, the shear stress was linearly increased from 0.01 to 0.9 Pa within 4 min.

### 2.3. Evaluation of the Antimicrobial Activity

Due to the manifold biofunctional activities (Figure 1) an unambiguous evaluation regarding all these functionalities is challenging. Within the scope of this study, we focused on the antibacterial properties. Therefore, the inhibitory potential of the WPC_biofunct._ on the growth of *Escherichia coli K12* (No.: 423, DSMZ, Braunschweig, Germany) was assessed. For comparison purposes, a commercial WPC80 powder (Sachsenmilch Leppersdorf GmbH, Wachau, Germany) and a purified and freeze-dried Lf powder (Tatura Milk Industries Pty. Ltd., Tatura, Australia) was used. 

Since these reference products were purchased as dried powders, the WPC_biofunct._ was freeze-dried in a first step (dryer: Alpha 2-4 LD, Martin Christ GmbH, Osterode am Harz, Germany). Subsequently, the inhibitory potential was assessed by incubating *E. coli K12* bacteria in 96-well microtiter plates. Each well was filled with 200 µL lysogeny broth (LB) media that contained defined concentrations (10–180 g·L^−1^) of the WPC_biofunct._ or a corresponding concentration of the reference products. The incubation was performed for 8 h in lysogeny broth (LB) media at 37 °C. During this incubation time, the bacterial growth was monitored by measuring the optical density at 620 nm (OD620 nm) in a plate reader every 20 min (Infinite 200 Pro, Tecan AG, Mannedorf, Switzerland). 

Based on the OD_620nm_ growth curves were derived. For the calculation of the growth inhibition, an incubated sample with pure LB media (without WPC_biofunct._, WPC80 or Lf powder) served as reference. For the calculation of the inhibitory potential the OD_620nm_ after 120 min incubation time, marking the turning point of the exponential growth phase, was used (Equation (7)):

(7) $$\mathrm{Growth}\;\mathrm{inhibition}=\frac{{\mathrm{OD}}_{620\;\mathrm{nm};\;120\;\min}}{{\mathrm{OD}}_{620\;\mathrm{nm};\;120\;\min\_\mathrm{reference}}}$$

### 2.4. Data Processing and Calculations

Data collection and processing was conducted with Excel (Version Professional Plus 2019; Microsoft Corp., Redmond, WA, USA). The SigmaPlot software (Version 12.5; Systat Software Inc., San Jose, CA, USA) was used for the creation of diagrams. The filtration experiments for the membrane selection were conducted in triplicate for each membrane, which resulted in a good reproducibility of the experimental design (Section 3.1). Therefore, we decided to carry out the resource-intensive concentration run in single determination. All analytical measurements (proteins, dry matter, viscosity) and subsequent experiments (antimicrobial activity assays) were performed in triplicate. The standard deviation is given next to the mean value as a measure of the range of variation. For clarification, the reference for the stated standard deviation has been included in the respective figure and table captions.

## 3. Results and Discussion

### 3.1. Membrane Selection

The selection of an appropriate membrane is of fundamental importance for a successful filtration, which is characterized by a high permeate flux (J_Permeate_) and a high retention of biofunctional proteins (Ret_Lf/IgG_). Furthermore, the membrane should be permeable for all further dry matter components (e.g., minerals, sugars, peptides and smaller whey proteins). In the dairy sector, ultra- and microfiltration membranes are typically used for protein fractionation (e.g., fractionation of casein and whey protein [38]) or protein concentration (e.g., WPC production [6]). In accordance with the membrane specifications, they are permeable for lactose (the major dry matter component of whey) and minerals. Consequently, the membrane selection focused on the transmission of the protein fraction.

**Figure 2:**
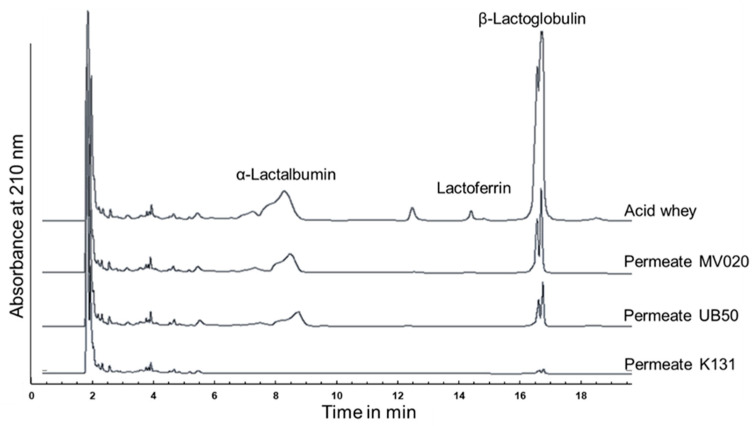
HPLC-Chromatogram of whey proteins in acid whey (feed) and in the permeates of different ultra- and microfiltration membranes (nominal pore sizes: MV020 = 200 nm; UB50 = 30 nm; molecular weight cut off: K131 = 10 kDa). The data refer to samples at filtration start (t = 5 min).

Figure 2 shows HPLC-chromatograms of the feed (acid whey) and permeates of the tested membranes. All membranes showed full retention for Lf since no peak could be detected in the permeate. In contrast to Lf, the whey proteins α-La and β-Lg partially permeated the MV020 (nominal pore size = 200 nm) and UB50 (nominal pore size = 30 nm) membranes, while they were fully retained by the K131 (MWCO = 10 kDa) membrane. At filtration start (t = 5 min) a Tr_Protein_ of 17 ± 4% was measured for the MV020 membrane, while the UB50 membrane showed a slightly lower Tr_Protein_ of 12 ± 2% (Table 1). With progressing time, the Tr_Protein_ decreased to 9 ± 3% (MV020), or respectively 4 ± 1% (UB50). This decrease originates from the formation of a deposit layer, a well-described phenomenon in membrane applications that affects the retention and flux characteristics [39]. Based on concentration polarization and absorption effects this layer is formed by retained components on the membrane’s surface. When a cross-flow filtration is conducted in equilibrium mode (constant feed composition), a steady state is reached after a certain time, where the thickness of the deposit layer, the Tr_Protein_ and the J_Permeate_ remain constant. Table 1 presents the J_Permeate_ at steady state (t = 100), showing a large difference between the K131 (J_Permeate_ = 14.1 ± 0.6 L·m^−2^·h^−1^) and the other membranes (MV020: J_Permeate_ = 38.6 ± 0.9 L·m^−2^·h^−1^; UB50: J_Permeate_ = 39.6 ± 0.8 L·m^−2^·h^−1^).

Considering the measured deviations for J_Permeate_ and Tr_Protein_, the reproducibility of the experimental design is good (Table 1). For all membranes, the deviations in J_Permeate_ at the end of filtration were less than 5%. The transmission values showed a slightly higher range of variation, but the differences between the membranes can be clearly recognized. Summarizing these results, all tested membranes allowed the retention and concentration of the biofunctional proteins Lf and IgG (IgG was not measured during the membrane tests, but the Ret_IgG_ must be higher than Ret_Lf_ due to the increased molecular weight). However, focusing on a maximum CF_Lf/IgG_ the MV020 and UB50 membranes seem more appropriate due to the higher Tr_Protein_ and J_Permeate_. Therefore, we selected the UB50 membrane for all further experiments.

### 3.2. Impact of Transmembrane Pressure on Flux and Protein Transmission

As seen in the previous section, the deposit layer has a notable influence on the transmission and flux behavior. While the impact of the filtration parameters on the membrane itself is limited, they can largely affect the intensity of deposit layer formation. As described in Section 2.1.3 the variation of Δp_TM_ offers the largest optimization potential. 

**Figure 3:**
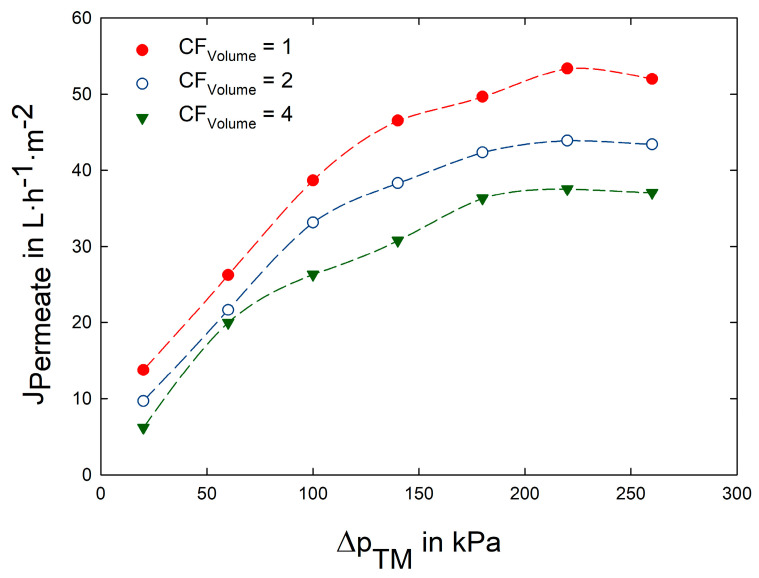
Permeate flux (J_Permeate_) as a function of transmembrane pressure (Δp_TM_) during the filtration (ϑ = 8 °C, nominal pore size: 30 nm) of acid whey (pH = 4.5).

In Figure 3 the J_Permeate_ as a function of Δp_TM_ is presented. The different symbols represent the flux values at varying concentration factors (CF_Volume_ = 1, 2 and 4). At all concentration factors, a similar curve progression was observed, whereby an increasing Δp_TM_ resulted in a higher J_Permeate_. This increase was observed until Δp_TM_ values of 180–220 kDa were reached, where the curve flattened and no further increase of J_Permeate_ was observed. Consequently, the critical Δp_TM_ for the concentration of acid whey using the UB50 membrane in spiral wound configuration (at w = 0.58 m·s^−1^ and ϑ = 8 °C) is settled in this range.

**Figure 4:**
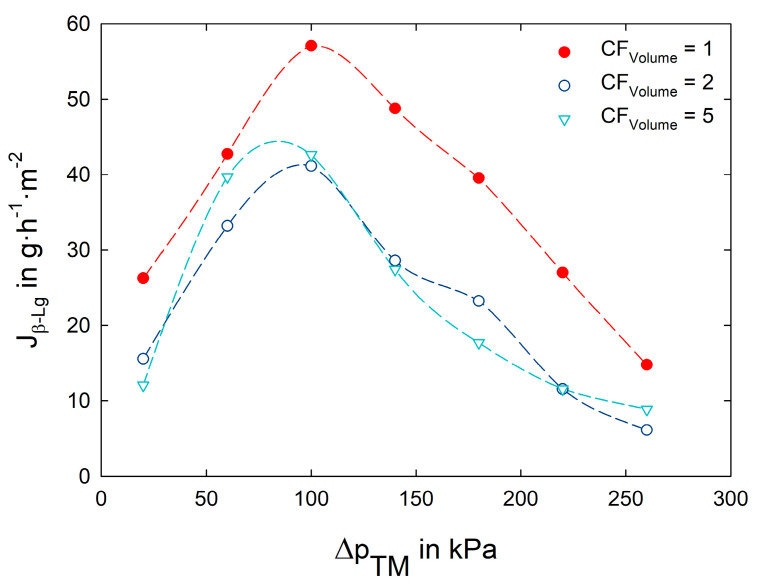
Mass flux of the whey protein β-lactoglobulin (J_β-Lg_) as a function of transmembrane pressure (Δp_TM_) during the filtration (ϑ = 8 °C, nominal pore size: 30 nm) of acid whey (pH = 4.5).

Figure 4 shows the mass flux of β-lactoglobulin (J_β-Lg_) as a combined measure of the J_Permeate_ and Tr_β-Lg_ (Equation (6)). The parameter optimization was oriented towards the major whey protein β-Lg, since it contributes with 50% to the overall protein content in whey and it showed a reduced transmission compared to α-La (at Δp_TM_ = 100 kPa and CF_Volume_ = 1: Tr_α-La_ = 56%; Tr_β-Lg_ = 36%). For a targeted enrichment of the biofunctional proteins Lf and IgG a high mass flux through the membrane of highly abundant proteins (e.g., β-Lg) is endeavored. While the J_β-Lg_ decreased with increasing CF_Volume_ (because of decreasing J_Permeate_ and Tr_β-Lg_ values), a definite mass flux maximum was observed in the range between Δp_TM_ = 90 and 100 kPa. At lower Δp_TM_ the J_Permeate_ was insufficient (Figure 3), while at higher values the Tr_β-Lg_ decreased due to an intensified deposit layer formation. At filtration start (CF_Volume_ = 1) a Tr_β-Lg_ of 54% was recorded at Δp_TM_ = 20 kPa, showing an almost linear decrease down to 7% at Δp_TM_ = 260 kPa.

Concerning the selective enrichment of Lf and IgG, a low Δp_TM_ would be desirable since the transmission of the smaller and non-functional whey proteins is increased. On the other hand, the J_Permeate_ is reduced at low-pressure values, resulting in prolonged filtration times. To find a compromise between a highly selective and an efficient filtration, the in Figure 4 depicted maximum of J_β-Lactoglobulin_ at a Δp_TM_ of 100 kPa was used for all further experminets. Based on this parameter setting (Δp_TM_ = 100 kPa, ϑ = 8 °C, w = 0.58 m·s^−1^), the acid whey concentration was performed, as described in the following section.

### 3.3. Enrichment of Lactoferrin and Immunoglobulin G

In the final enrichment experiment, a more than 30-fold volume reduction was achieved. The filtration in concentration mode had to be stopped when a critical feed volume of approximately 26 L was reached and air was soaked in by the feed pump. Consequently, even higher concentration factors could be realized with a sufficient feed volume.

**Figure 5:**
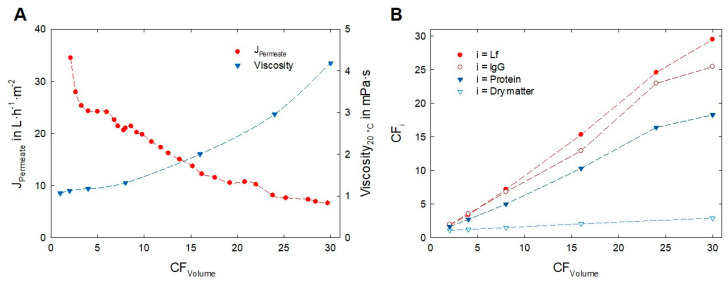
Permeate flux (J_Permeate_) and retentate viscosity during the concentration of acid whey (**A**). (**B**) depicts the increase in concentration factor of whey proteins and dry matter (CF_i_) as a function of the volume concentration factor (CF_Volume_). The cross-flow filtration (ϑ = 8 °C, Δp_TM_ = 100 kPa, nominal pore size = 30 nm, active area: 5.4 m^2^) was conducted for 200 min using clarified acid whey (pH = 4.5) as feed.

The red line in Figure 5A shows the decrease of J_Permeate_ from 35 L·h^−1^·m^−2^ (at CF = 1) to 6.5 L·h^−1^·m^−2^ (at CF*_Volume_* > 30). The flux curve showed a steep decrease directly after filtration start (formation of the initial deposit layer), which flattened within the first 20 min of filtration. After this initial phase, an approximately linear correlation between J_Permeate_ and CF_Volume_ was observed. At a CF_Volume_ of 23, the flux dropped below 10 L·h^−1^·m^−2^ and converged to 6 L·h^−1^·m^−2^ in the final phase. The rheological measurements of the whey and retentate samples showed the behavior of a Newtonian fluid. The viscosity (Figure 5A, blue line) exhibited an exponential dependency on CF_Volume_. The measured viscosities are in good agreement with the results of Morison and Mackay [40] who showed that up to a whey protein content of 150 g·L^−1^ the relation between viscosity and solutes volume can be described by a higher order form of Einstein’s equation.

In Table 2 the composition of the whey and retentate is compared. The crude protein content increased from 7.0 ± 0.4 g·L^−1^ to 127.2 ± 0.7 g·L^−1^, representing a CF_Protein_ of 18. Based on dry matter content the final retentate contained 70% of crude protein (WPC70). Considering the biofunctional protein fraction, an almost 30-fold enrichment of Lf and a 25-fold enrichment of IgG was achieved. Comparing the Lf and IgG amount in the whey and biofunctional retentate (WPC70_biofunct._) a recovery of 97% for Lf and 83% for IgG was measured.

Figure 5B shows the selective enrichment of these functional proteins, compared to the crude protein and dry matter content. As was already seen in the optimization runs (Section 3.1), the transmission of α-La was notably higher compared to β-Lg. After the initial filtration phase, which was comparable with the data of Figure 4, the Tr_α-La_ remained constant between 30 and 35% over the entire filtration time, while the Tr_β-Lg_ was settled between 3 and 5%. Therefore, β-Lg remained the most abundant protein, but the ratio of the biofunctional proteins to overall protein increased. This proportional shift becomes more obvious when the dry matter content is used as reference, as it is relevant for dried products. Based on dry matter, the share of Lf and IgG increased almost 10-fold from 0.66% (in acid whey) to 6.18% (in the retentate). For a higher selectivity between functional and crude protein, the Tr_β-Lg_ must be increased. Targeting on the aggregation behavior might be a promising approach [41] since the molecular weight of α-La (14 kDa) and β-Lg (18 kDa) is within a comparable range. Through diafiltration, the milieu conditions might be modified to suppress the aggregation potential, resulting in an enhanced permeation of non-aggregated β-Lg monomers. However, for the health-promoting properties, the absolute concentration of functional proteins is of far greater importance than relative ratios. 

Considering the in Section 1 derived benchmark for the daily Lf intake (>200 mg), this would correspond to a volume of approximately 40 mL in liquid form, or an amount of 8 g in dried powder form. These small amounts of the final WPC70_biofunct._ represent such a small amount that they can be easily integrated into the daily food intake. In addition, the retentate might serve as biofunctional ingredient for further product developments.

### 3.4. Evaluation of Biofunctional Properties—Antibacterial Activity

A comprehensive assessment of the biofunctional properties of the produced retentate, referred to as WPC70_biofunct._, is challenging due to the manifold efficacy spectrum of the concentrated proteins (Figure 1). Therefore, we confine the following explanations of the evaluation of the antibacterial activity.

**Figure 6:**
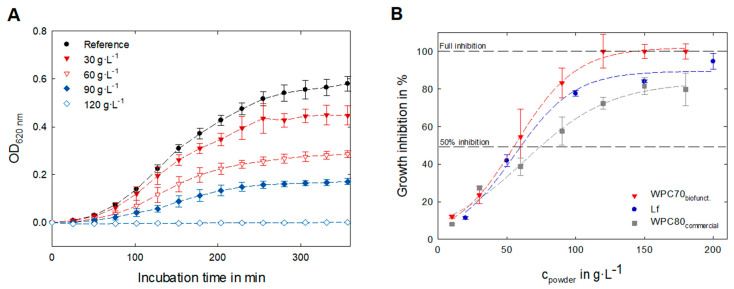
Bacterial growth of E. coli K12 in LB media at 37 °C depending on the WPC70_biofunct_. concentration (**A**). Dose dependent growth inhibition of the functional retentate (WPC70_biofunct._) and commercial reference samples (pure lactoferrin powder and WPC80_commercial_; (**B**). The presented standard deviation refers to the antimicrobial assay conducted as triplicate using the same batch of whey protein powders.

In Figure 6A the growth inhibition of WPC70_biofunct._ on *E. coli K12* bacteria is shown. Already small amounts (in the range of 5–30 g·L^−1^) of the freeze-dried whey powder resulted in unequivocal deviations from the reference sample. At higher concentrations (30–100 g·L^−1^), the growth inhibition became more prominent up to concentrations >120 g·L^−1^, where a full growth inhibition was observed (within the 8 h observation period).

Based on these concentration-dependent growth curves, a dose–response relationship for WPC70_biofunct_ was derived (red line, Figure 6B). Furthermore, the dose–response relationship for a commercial WPC80 and a pure Lf powder is shown in Figure 6B. All tested powders showed an inhibitory potential on the growth of *E. coli K12*; however, only for the WPC70_biofunct._ was a full inhibition within the tested concentration range derived. The inhibitory capacity of the reference powders was lower, compared to the Lf and IgG enriched powder. A conclusive measure, therefore, is the inhibitory capacity 50 (IC_50_), which describes the amount of an antibacterial ingredient that is necessary for a 50% growth inhibition (Figure 6B and Table 3).

Being premised on the overall powder concentration, the IC_50_ for the WPC70_biofunct._ Is 55.6 g·L^−1^, while for pure Lf and WPC80_commercial_ notable larger amounts were required for a comparable effect. The low ratio of antimicrobial active proteins could explain the reduced growth inhibition of the WPC80_commercial_ Interesting is the decreased inhibitory potential of pure Lf, since the absolute Lf concentration is 35-fold higher than in the corresponding WPC70_biofunct_. sample. This means that despite the fact that the absolute concentration of the biofunctional protein Lf is higher in the purified Lf sample, the observed antibacterial effects are lower than in the WPC70_biofunct._ powder. Comparing the concentration of biofunctional proteins at the IC_50_, the amount of Lf and IgG is only 3.4 g·L^−1^ in the WPC70_biofunct._, while 60.7 g·L^−1^ of the purified Lf sample is needed for a similar effect. Synergistic effects and interactions with other whey ingredients (e.g., proteins or peptides) are an explanation for this potent activity of the Lf- and IgG-enriched biofunctional retentate.

## 4. Conclusions

By means of cross-flow ultrafiltration, a selective enrichment of the biofunctional proteins lactoferrin and immunoglobulin G from acid whey is possible. Under optimized filtration conditions (ϑ = 8 °C, Δp_TM_ = 100 kPa, w = 0.58 m·s^−1^, membrane: Trisep UB50, nominal pore size: 30 nm) a more than 30-fold concentration was achieved in the experimental runs. Since the criterion for the concentration stop was the limited feed volume, even higher concentration factors can be reached with this set of parameters until a complete membrane blockage occurs.

A comparison with commercial supplements and literature data revealed that due to the elevated concentration of functional proteins (c_Lf_ = 5.1 g·L^−1^; c_IgG_ = 6.1 g·L^−1^) a daily intake of 40 mL might be sufficient for health-promoting effects. In contrast to reference products (e.g., purified Lf or colostrum-based supplements), the WPC70_biofunct._ was produced from a highly abundant, and so-far-unexploited, by-product of dairy processing. Furthermore, the produced retentate is rich in both high-value proteins and contains additional bioactive ingredients. The assessment of the antibacterial properties showed a full antimicrobial growth inhibition against *E. coli K12* bacteria at a WPC70_biofunct._ concentration of 120 g·L^−1^. Moreover, the functional retentate showed a higher antibacterial activity than pure lactoferrin powder, indicating that synergistic effects within the manifold retentate ingredients might amplify the biofunctional effects.

## Figures and Tables

**Table 1:** Comparison of three different ultra- and microfiltration membranes (nominal pore sizes: MV020 = 200 nm; UB50 = 30 nm; molecular weight cut off: K131 = 10 kDa) regarding flux and retention characteristics. The presented lactoferrin retention (Ret_Lf_) and protein transmission values (Tr_Protein_) refer to the situation at filtration start (t = 5 min), while the flux values (J_Permeate_) refer to steady state conditions (t = 100 min; filtration in equilibrium mode). The stated standard deviations refer to filtration runs carried out as triplicate for each membrane.

Membrane	Ret_Lf_ in %	Tr_Protein_ in %	J_Permeate_ in L·m^−2^·h^−1^
MV020	>95%	17 ± 4	38.6 ± 0.9
UB50	>95%	12 ± 2	39.6 ± 0.8
K131	>95%	<5	14.1 ± 0.6

**Table 2:** Composition of acid whey and the final retentate, designated as WPC70_biofunct._ The presented standard deviation refers to the analytical method measured as triplicate.

		Acid Whey	WPC70_biofunct._	CF_Volume/i_
Volume	in L	800	26	×30.8
Viscosity_20°C_	in mPa·s	1.063 ± 0.003	4.19 ± 0.06	
Dry matter	in g·L^−1^	62.7 ± 0.7	181.7 ± 0.8	×2.90
Protein	in g·L^−1^	7.0 ± 0.4	127.2 ± 0.7	×18.2
Lf	in g·L^−1^	0.17 ± 0.01	5.1 ± 0.1	×29.5
IgG	in g·L^−1^	0.24 ± 0.03	6.13 ± 0.44	×25.3
α-La	in g·L^−1^	0.89 ± 0.05	13.10 ± 0.23	×14.8
ß-Lg	in g·L^−1^	3.71 ± 0.18	99.8 ± 1.4	×26.9

**Table 3:** Comparison of the inhibitory concentration 50 (IC_50_) of the whey protein concentrate with increased lactoferrin (Lf) concentration (WPC70_biofunct._) and a commercial Lf and and whey protein concentrate (WPC80_commercial_) powder. The presented data refer to *E. coli*, incubated in LB-media at 37 °C.

	IC_50_	
	c_powder_ in g∙L^−1^	c_Lf_ in g∙L^−1^
WPC70_biofunct._	55.6	1.5
Lf	60.7	58.9
WPC80_commercial_	75.5	~0.6 ^1^

^1^ Assumed value based on a ratio of 1% Lf on total protein.

## Data Availability

Data is contained within the article or supplementary material.

## Supplementary Materials

The following supporting information can be downloaded at: https://www.mdpi.com/article/10.3390/foods12112163/s1. Figure S1: Piping and instrumentation diagram of the pilot scale filtration plant.
