# Supplementary figures and images for: Enrichment of Lactoferrin and Immunoglobulin G from Acid Whey by Cross-Flow Filtration
Source: Foods. 2023 May 26;12(11):2163. doi: 10.3390/foods12112163 (PMC10252284; doi:10.3390/foods12112163)

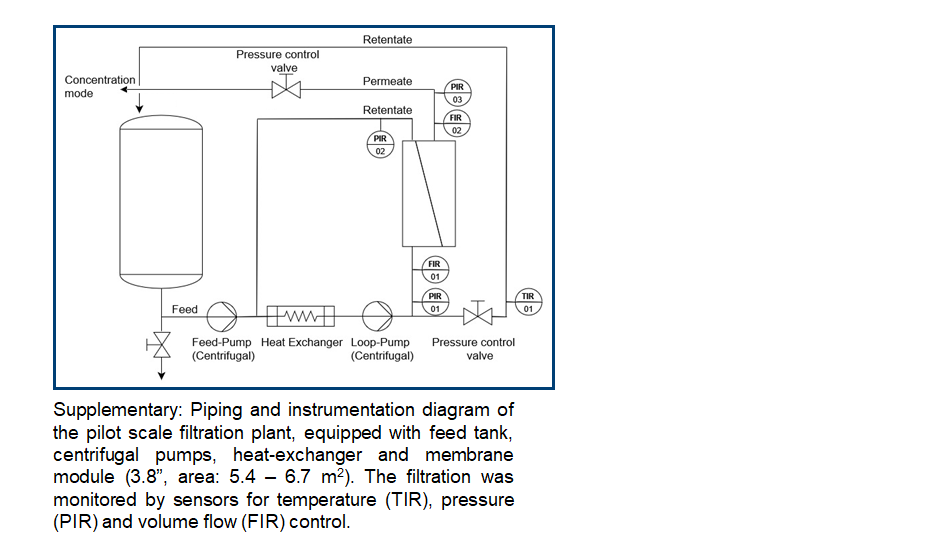

Supplement: Supplementary file 1 [file foods-12-02163-s001.zip › foods-2392454-Supplementary Figure S1.png]
